# Productivity Losses Due to Diabetes in Urban Rural China

**DOI:** 10.3390/ijerph19105873

**Published:** 2022-05-12

**Authors:** Hongying Hao, Stephen Nicholas, Lizheng Xu, Anli Leng, Jingjie Sun, Zhiyan Han

**Affiliations:** 1 Graduate School, Shandong First Medical University & Shandong Academy of Medical Sciences, 6699 Qingdao Road, Jinan 250013, China; haohongying@sdfmu.edu.cn; 2 Centre for Health Management and Policy Research, School of Public Health, Cheeloo College of Medicine, Shandong University, Jinan 250012, China; 3 NHC Key Lab of Health Economics and Policy Research, Shandong University, Jinan 250012, China; 4 Australian National Institute of Management and Commerce, 1 Central Avenue Australian Technology Park, Eveleigh, NSW 2015, Australia; stephen.nicholas@newcastle.edu.au; 5 Guangdong Institute for International Strategies, Guangdong University of Foreign Studies, 2 Baiyun North Avenue, Guangzhou 510420, China; 6 School of Economics and School of Management, Tianjin Normal University, No. 339 Binshui West Avenue, Tianjin 300387, China; 7 Newcastle Business School, University of Newcastle, Newcastle, NSW 2308, Australia; 8 School of Medical Science, The University of New South Wales, Kensington, NSW 2052, Australia; lizheng.xu@unsw.edu.au; 9 The George Institute for Global Health, Newtown, NSW 2042, Australia; 10 School of Political Science and Public Administration, Institute of Governance, Shandong University, 72 Binhai Road, Qingdao 266237, China; lenganli@sdu.edu.cn; 11 Shandong Health Commission Medical Management Service Center, Jinan 250012, China; sunjingjie@shandong.cn; 12 School of Health Care Security, Shandong First Medical University & Shandong Academy of Medical Sciences, 6699 Qingdao Road, Jinan 250013, China

**Keywords:** productivity losses, premature deaths, diabetes

## Abstract

Background: Productivity losses due to diabetes are increasing in China, but research about the impact of diabetes on productivity in urban and rural areas requires further in-depth study. This article provides the first estimate of the cost of productivity losses attributed to diabetes in individuals 20–69 years old in urban and rural areas of China. Methods: The human capital approach is employed to measure the productivity losses attributed to absenteeism, presenteeism, labor force dropout, and premature deaths due to diabetes of the 20–69-year-old population of males and females in urban and rural areas of China. Based on the life table modelling, we calculate the years of potential life lost and working years of life lost of people with diabetes. Results: In 2017, we estimated that there were 100.46 million people with diabetes, with the total cost of productivity losses being USD 613.60 billion, comprising USD 326.40 billion from labor force dropout, USD 186.34 billion from premature death, USD 97.71 billion from absenteeism, and USD 27.04 billion from presenteeism. Productivity loss was greater in urban (USD 490.79 billion) than rural areas (USD 122.81 billion), with urban presenteeism (USD 2.54 billion) greater than rural presenteeism (USD 608.55 million); urban absenteeism (USD 79.10 billion) greater than rural absenteeism (USD 18.61 billion); urban labor force dropout (USD 261.24 billion) greater than rural labor force dropout (USD 65.15 billion); and urban premature death (USD 147.90 billion) greater than rural premature death (USD 38.44 billion). Conclusions: Diabetes has a large and significant negative impact on productivity in urban and rural China. Productivity loss is significantly higher in urban versus rural regions. Further investment is required in the prevention, diagnosis, and control of diabetes in under-resourced health services in rural locations as well as in urban areas, where most diabetes cases reside. Specifically, targeted and effective diabetes prevention and management actions are urgently required.

## 1. Background

Diabetes mellitus is a major chronic disease [1,2], significantly affecting individuals’ ability to participant in the labor force and reducing the productivity of working people with diabetes [3]. Globally, an estimated 463 million people suffered from diabetes in 2019, 56% of whom were undiagnosed [4,5], and one in four diagnosed diabetes sufferers worldwide came from China [4]. There are significant disparities in the prevalence and mortality rates of diabetes between urban and rural locations, with the prevalence of diabetes being 8.15% in urban areas and 4.13% in rural areas among the 30–79-year-old population in 2004–2008 [6], 11.4% in urban locations and 8.2% in rural locations among those 20 years or older in 2008 [7], and 14.3% in urban locations and 10.3% in rural locations among those 18 years or older in 2010 [8]. The prevalence of diabetes also varied by gender, with 7.1% for men in the 20–59 age group and 4.67% for women in the 20–49 age group in 2017 suffering from diabetes [4,9]. With an adjusted prevalence rate of diabetes of 9.2% among the 20–79 age group, it was estimated that people with diabetes could increase by 140.45 million by 2030 and increase again by 147.24 million by 2045 in China [5]. Industrialization, urbanization, population aging, and changes in the ecological environment and lifestyle, including obesity, diet, and physical activity [2], have led to the increasing diabetes prevalence rates and different rates in urban and rural areas. Importantly, the productivity losses due to diabetes differ significantly between urban and rural areas.

Inadequate scientific and normative diabetes prevention and management, the risk of diabetes complications, and diabetes related disabilities and premature deaths pose significant burdens on people with diabetes, their families, and the health care system [6]. The burden of chronic non-communicable diseases, especially diabetes, is an issue of increasing concern to governments. Many studies have researched the impact of diabetes on productivity losses at the global level [10] and country level [11,12,13,14,15,16,17,18,19]. Given the significant productivity losses due to diabetes in other countries, there are surprisingly few studies about diabetes productivity losses in China. The productivity costs of diabetes in China are likely to be nontrivial [9], imposing an economic burden on individuals, employers, and the government through reduced earnings, work effort, tax revenue, and gross domestic product (GDP).

The International Diabetes Federation (IDF) estimated that the diabetes cost in China was approximately USD 109.8 billion in 2017 [4] and USD 109.01 billion in 2019 [5], which only estimated the ‘direct’ costs of diabetes based on healthcare expenditure but did not include the costs of productivity losses from absenteeism, presenteeism, and premature mortality. Hird et al. [9] estimated that productivity adjusted life years (PALYs) attributed to diabetes for Chinese women aged 20–49 years and for Chinese men aged 20–59 years was USD 2.6 trillion, comprising USD 1.6 trillion for labor force dropout, USD 640.8 billion for premature deaths, USD 232.9 billion for absenteeism, and USD 108.7 billion for presenteeism. However, previous studies did not estimate separately the urban and rural productivity losses despite urban–rural differences in diabetes prevalence and mortality being significant in China. The mortality of diabetes sufferers in rural areas was higher than in urban areas, whereas the prevalence of diabetes in urban areas was higher than in rural areas [6,7,8]. These urban–rural differences call for an assessment of whether there were significant urban–rural productivity loss differences. Our study provides the first estimates of the cost of productivity losses due to premature deaths, absenteeism, presenteeism, and labor force dropout for 20–69-year-old people with diabetes in rural China and the first comparison of urban and rural productivity losses due to diabetes. Our findings will inform health policy interventions and the allocation of health resources in urban and rural areas.

## 2. Methods

### 2.1. Study Design and Setting

We use the human capital approach to estimate the value of productivity losses attributed to diabetes, due to labor force dropout, absenteeism, presenteeism, and premature deaths. Operationalized for use in public health by Weisbrod [20], the human capital approach to productivity calculates the present value of the sum of earnings and the imputed value of household production over an individual’s lifetime, discounted for diabetes [21]. Productivity loss takes all working hours lost due to diabetes health problems and related treatments and multiplies that by the gross hourly wage [20]. Diabetes covers all types of diabetes, including type 1 diabetes, type 2 diabetes, gestational diabetes, other types of diabetes, and diabetes complications.

Absenteeism is being away from work. There are no estimates of absenteeism due to diabetes in China. We have proxied Chinese absenteeism rates by using data from other countries. In 2014–2016, the average absent days per year due to diabetes in America was 1.7 days, without any distinction between rural and urban location or gender [15]. Furthermore, an overview of absenteeism due to diabetes in a set of low-middle income countries workers was estimated to be 1.9 days for men and 10.2 days for women per year [10]. Using low-income countries’ absenteeism rates, we multiplied China’s lost hours due to absenteeism by the average wage per hour [22]. In China, the work year was 246 days in 2017 and the weekly working hours of employed persons was 46.2 h [23], based on 5 working days per week with average working time of 9.24 h per day. With a USD 2.37 urban wage per hour, and a rural wage per hour of USD 0.87, the cost of lost productivity for urban men was 1.9 × 9.24 × USD 2.37 or USD 41.61 per man per year and 10.2 × 9.24 × USD 2.37 or USD 223.37 per urban woman per year in urban locations; and 1.9 × 9.24 × USD 0.87 or USD 15.27 per rural man per year and 10.2 × 9.24 × USD 0.87 or USD 82.00 per rural woman per year.

Presenteeism is coming to work while being unwell, resulting in a shortfall in work output. In the absence of estimates of presenteeism for diabetes patients in China, we used Boomer et al.’s [10] estimate of the shortfall in productivity due to diabetes-associated presenteeism of 0.6% for men and 1.0% for women from low-middle income countries. Per patient productivity losses were estimated by multiplying the average urban (USD 5388.36) and rural (USD 1993.08) resident’s disposable income by 0.6% for men and 1.0% for women, which was then multiplied by the total number of diabetes patients.

The labor force dropout rate is the percentage of workers with diabetes-related disabilities exiting the workplace. There are no studies of labor force dropout rates for diabetes sufferers in China. Using estimates from a global survey of cost-of-illness due to diabetes, we used a dropout rate of 7% for women and 5.2% for men among diabetes sufferers aged 20–29 years old and 12.8% in women and 8.3% in men over 40 years old [10]. For the 30–39 age group, we estimated the dropout rate. In 2017, the unemployment rate for the 30–39 age group without diabetes was 3.9% in China [23]. Based on a U.S. logistic regression study [15] that showed that the labor force dropout rate of diabetes was 3.1% higher than the dropout rate of the population without diabetes, we estimated the 30–39 age group dropout rate as 3.9% × (1 + 0.0031) or 4.02%. Earnings per year, per resident was resident disposable income RMB 36396.19 (USD 5388.36) in urban and RMB 13462.43 (USD 1993.08) in rural locations in 2017 [24]. Finally, premature deaths were obtained by the number in each age and gender-specific group in the 20–69 age group population multiplied by the corresponding mortality rate of diabetes in the corresponding age and gender group of diabetes.

We estimated life expectancy, years of potential life lost (YPLL), and working years of potential life lost (WYLL) for 2017. Life table modelling was constructed to obtain the life expectancy of people in urban and rural regions for 5-year age band-specific mortality rate data for the Chinese population in 2017 using MORTPAK4.3 software. We assumed that the rate for a 5-year age group applied to people in the midpoint of that age band.

### 2.2. Data Sources

We calculated the present value of people with diabetes earnings in their expected life based on the years of lost disposable income by urban and rural residents in 2017. All costs were estimated in Chinese RMB before being converted to U.S. dollars at the 2017 exchange rate, USD–RMB6.8 [25]. The demographic data were obtained from the China Statistical Yearbook 2018 [24] and the China Population and Employment Statistical Yearbook 2018 [23], including population size stratified by age group (5-year bands), gender, and rural and urban location. The Yearbook data were based on a representative sample of the national population in 2017, with a 0.824‰ sampling ratio. Age and gender-specific prevalence of diabetes were estimated by age and gender from the 2017 International Diabetes Federation Diabetes Atlas [4]. The mortality data of diabetes were provided by the China Health Statistics Yearbook 2018 [26], which reports death of residents from illness and injury. The mortality data were used to construct the life table modelling by MORTPAK 4.3 software. The data of resident disposable income were obtained from the China Statistics Yearbook 2018.

Since 2012, over 97% of the population has been covered by health insurance [27]. The main pension system was the enterprise staff basic endowment insurance for employees in urban areas and the basic endowment insurance for residents in urban and rural areas. The enterprise staff basic endowment insurance for employee covered employed and retired urban workers; basic endowment insurance for residents in urban and rural areas covered the urban unemployed residents and rural residents. The retirement age of the enterprise staff basic endowment insurance system was 60 years old for men, 55 years old for urban managerial women, and 50 years old for women workers. The retirement age was 60 years old for all the residents who participated in basic endowment insurance for residents in urban and rural areas. Much of the aging population continued to participate in the labor force. We define the 20–69 age group as the sample. We assumed the labor force participation rate of total population was also the rate of labor force participation of people with diabetes. The labor force participation rate of urban men was 46.2% in the 60–64 age group and 31.6% in the 65–69 age group; the labor force participation rate of urban women was 49.2% in the 50–54 age group, 33.4% in the 55–59 age group, 31.4% in the 60–64 age group, and 21.2% in the 65–69 age group. There was a higher rate of labor force participation in rural areas for both females and males. The labor force participation rate of rural men in the 60–64 and 65–69 age group was 84% and 74.4, respectively. The labor force participation rate of rural women in the 60–64 and 65–69 age group was 72.6% and 63.4%, respectively [28]. Given differences between the urban and rural endowment insurance funding modalities and the pension rate [26], urban workers received on average ¥ 2300 (USD 340.51) per person per month in 2017 and rural workers received on average ¥ 120 (USD 17.77) per person per month [23].

### 2.3. Statistical Analysis

Microsoft Excel 2016 (2109 Build 16.0.14430.20256) was used to calculate the productivity losses and to estimate the cost of productivity losses due to diabetes. MORTPAK4.3 software was used to estimate life expectancy.

## 3. Results

### 3.1. The Prevalence and Mortality of Diabetes in China

In 2017, the average prevalence of diabetes for adults aged 20–69 years was 10.14%, comprising 10.21% in urban areas and 10.04% in rural locations, with 61.69 million people with diabetes in urban locations and 38.77 million in rural areas or a total of 100.46 million people with diabetes. From Table 1, the prevalence of diabetes in urban males was 11.77%, which was significantly higher than for urban females (8.60%), whereas Table 2 estimates that there was no significant difference in rural male (10.52%) and rural female (9.53%) diabetes prevalence rates.

We estimated the YPLL, WYLL, and productivity losses based on age and gender-specific mortality from diabetes in urban and rural areas. As shown in Table 1 and Table 2, the aggregate mortality of diabetes in the 20–69 age group was 8.37/100,000 in urban locations and 9.93/100,000 in rural locations, with the mortality of urban males (9.75/100,000) significantly higher than urban females (6.93/100,000), but the mortality rate was similar for rural males (9.98/100,000) and rural females (9.87/100,000).

### 3.2. Premature Deaths and Work Years Lost Due to Diabetes

As shown in Table 3 and Table 4, there were about 88.88 thousand (38.33 thousand in urban and 50.55 thousand in rural locations) premature deaths attributed to diabetes in the 20–69 age group. From Table 3 and Table 4, people who died prematurely from diabetes in urban locations was significantly higher than those who died of diabetes in rural areas (χ^2^ = 636.348, *p* < 0.05).

From Table 3 and Table 4, the total YPLL was 2.5 million years, with 1.43 million YPLL in urban areas and 1.07 million YPLL in rural areas. YPLL for diabetes in urban areas (1.43 million) was higher than in rural areas (1.07 million). YPLL by urban males (0.99 million) was higher than rural males (0.58 million) and YPLL by rural females (0.49 million, 46.16%) was higher than urban females (0.44 million, 30.31%). Male YPLL in the 65–69 age group was the highest for all age groups, whether in urban or rural areas. As shown Table 3 and Table 4, the WYLL was 1.05 million, comprising 0.58 million in urban locations and 0.47 million in rural locations. Whether residing in urban or rural locations, the WYLL for males was absolutely higher than for females: for urban males it was WYLL 0.58 million (89.11%) versus 0.07 million (10.89%) WYLL for urban females and 0.28 million (59.81%) for rural males versus 0.19 million (40.19%) for rural females. The percentage of WYLL of the 50–54-year-old group was more than any other age group, whether from urban or rural areas.

### 3.3. Cost of Productivity Losses from Premature Death Due to Diabetes

Table 3 and Table 4 also estimated that the cost of productivity lost due to premature diabetes deaths was USD 9.84 billion (USD 7.71 billion in urban and USD 2.13 billion in rural locations). In urban areas, productivity losses of males (USD 5.34 billion) were higher than that of females (USD 2.37 billion). Similarly, the productivity losses of males in rural areas (USD 1.14 billion) were higher than that of rural females (USD 0.98 billion).

### 3.4. The Cost of Productivity Losses for Absenteeism, Presenteeism, and Labor Force Dropout Due to Diabetes

As estimated in Table 5, the cost of productivity losses from absenteeism was USD 65.71 billion (USD 22.61 billion in urban areas and USD 43.10 billion in rural areas); presenteeism cost was USD 2.71 billion (USD 1.78 billion in urban and USD 0.94 billion in rural areas); and labor force dropout due to diabetes costs were USD 268.52 billion (USD 207.28 billion and USD 61.24 billion). The total cost of productivity losses for absenteeism, presenteeism, and labor force dropout was USD 336.94 billion (USD 231.59 billion in urban areas and USD 105.28 billion in rural areas).

For 20–69-year-old people with diabetes, 69.01% of the value of total productivity losses occurred in urban areas, with the main reasons being labor force dropout (77.43%) and absenteeism (18.95%), which accounted for 96.38% of total urban productivity losses. In 2017, the cost of productivity losses for labor force dropout due to diabetes was USD 268.52 billion, followed by absenteeism (USD 65.71 billion), premature deaths (USD 9.84 billion), and presenteeism (USD 2.71 billion).

## 4. Discussion

Our study provides the first estimates of the cost of productivity losses due to premature deaths, absenteeism, presenteeism, and labor force dropout for the 20–69 age group of people with diabetes in rural China and the first comparison of urban and rural productivity losses due to diabetes. Our analysis revealed that there were significantly more people with diabetes in urban areas than rural areas. Both YPLL (1.43 million in urban areas and 1.06 million in rural areas) and WYLL (0.58 million in urban areas and 0.47 million in rural areas) were higher in urban locations than rural locations, which is consistent with urban and rural premature deaths from diabetes. Our estimate was 2.5 million years of total YPLL and Hird et al.’s estimate was 22.7 million years total YPLL. One reason for the YPLL differences between our estimates and Hird et al.’s was the different retirement ages. Hird et al. used 60 years old for men and 50 years old for women, but we provided a much more accurate retirement age of 60 years old for urban and rural men, rural women, and urban managerial women and 55 years old for urban managerial women and women workers. One obvious result of our more accurate retirement age data was that more females fell into the higher 50–60 prevalence of diabetes group than in Hird et al. Second, we used the China Health Statistic Book 2018 estimate of mortality of diabetes—88.88 thousand deaths—while Hird et al.’s estimated 7.29 million deaths. The number of deaths from diabetes was the main reason for the gap.

There were significant differences in the productivity losses between our estimates and Hird et al. and Boomer et al. [6]. We estimated that the costs of productivity losses for absenteeism, presenteeism, and the labor force dropout (USD 346.71 billion) accounted for 0.41% of GDP in China in 2017, which was higher than Bommer et al.’s [6] productivity loss estimate of USD 224.58 billion or 0.32% of GDP in 2015. Partly, the difference was due to differing assumptions and modelling and different sample sizes and populations. Our sample size was 100.46 million Chinese people with diabetes whereas Boomer et al.’s sample was the global diabetes population. Furthermore, Bommer et al. used annual wage data, whereas we used annual per capita income. Finally, mortality was higher in our study than Boomer et al.’s study, which led to a higher cost of productivity losses from premature death in our estimates. Our estimate of USD 346.71 billion productivity loss was significantly higher than Boomer et al.’s [6] USD 224.58 billion productivity loss estimate but significantly lower than Hird et al.’s [7] estimate of USD 2.6 trillion productivity loss. This difference was due to different estimation methods, wage data, and GDP per capita estimates. Hird et al. used the annual GDP per worker RMB 179,486 (USD 26,789), which was significantly higher than our urban RMB 36396.19 (USD5388.36) per capita disposable income and RMB 13462.43 (USD 1993.08) for rural locations [28]. Hird et al. projected temporal trends in GDP across the model time horizon using the OECD long-term GDP forecasts, with Hird et al.’s estimate of productivity adjusted life years (PALYs) lost to diabetes being 75.8 million years whereas our estimate is 2.5 million years. The number of diabetes deaths was also an important reason for the differences between studies.

Comparing previous estimates of the distribution of productivity losses from diabetes, our estimate of labor force dropout (77.42%) was higher than Boomer et al.’s [6] estimates (48.5%), but our premature death estimate (2.84%) was significantly lower than Boomer et al.’s (45.5%). Our estimated absenteeism (18.95%) was higher than Bommer et al.’s absenteeism (3.9%) and our presenteeism (0.78%) was lower than Bommer et al.’s presenteeism (2.1%) estimate. Our estimates of the distribution of productivity losses were broadly consistent with Hird et al. [9], whereas our labor force dropout rate of 77.42% was higher than Hird’s 62.1% and our 2.84% premature death was lower than Hird’s 24.7%. However, our estimate of absenteeism (18.95%) was higher, and our presenteeism rate (0.74%) was lower than Hird et al.’s [7].

We found that 69.02% of the cost of productivity losses occurred in urban areas, and the distribution of productivity losses due to workforce dropout, absenteeism, presenteeism, and premature death in rural and urban areas was different. In urban areas, the proportion of productivity losses for the labor force was up to 86.59%, and 57.02% in rural areas, and this was the majority of productivity losses in both urban and rural locations. There was a big gap between the proportions of productivity losses for absenteeism in urban and rural areas (USD 22.61 billion in urban areas and USD 40.13 billion in rural areas). The gap may relate to health literacy, awareness of diabetes, medication compliance, diets, social development levels, population size, urbanization, obesity, and lifestyle or habits. Other reasons may be economic and health-insurance related. There was a higher reimbursement ratio in Employee Basic Medical Insurance than Urban and Rural Residents’ Basic Medical Insurance. We recommend further health insurance reform to ensure that rural and urban diabetes sufferers benefit equally. Most of the productivity losses occurred in urban areas because urban areas were more highly developed than rural areas, and urban locations had more work opportunities. Per capita urban salaries were higher than wages in rural areas [23]. Coupled with urbanization, the surge in the urban labor force, mainly due to migrant workers, promoted urban diabetes rates.

High urban and rural rates of diabetes imply that the same policies and financial support for diabetes prevention and control might be applicable in both urban and rural areas. The significant gap in the level of diabetes management in urban communities compared to rural communities was further complicated by disparities in diabetes health management and health resources in urban versus rural areas. For example, urban locations had more health-related resources and better quality hospitals, medical staff, and medical equipment [28,29,30]. We recommend a targeted approach to the implementation of diabetes prevention and health management measures. For rural areas, we recommend increasing resources to appropriately fund primary health facilities; investing in training medical staff; and allocating funding for sustainable health education to improve health literacy. Because health literacy is key for diabetes prevention and health management of diabetes, we recommend the use of policy tools to attract excellent medical technical staff to improve diabetes education in both rural and urban areas. The targeted improvement in the provision of preventive health services, lifestyle programs for weight management, and health education and health management for diabetes programs are key recommendations for controlling diabetes. Specifically, we recommend healthy diet programs, regular physical activity, maintaining a normal body weight, scientific and health management of diabetes, and avoiding tobacco use, to help both urban and rural people with diabetes. Such lifestyle programs would require long-term and effective health education interventions.

There are some limitations and strengths to our research. The first limitation of our estimates was the absence of China-specific data or self-reported or individual data on absenteeism, presenteeism, and labor force dropout rates due to diabetes. Reliance on absenteeism, presenteeism, and labor force dropout rates from other countries may not reflect the exact rates for China, but rates from other countries provide the only alternative data for the missing and unknown Chinese data and this is consistent with other diabetes studies [10]. Second, our estimates omitted people with diabetes who were not employed and also some indirect productivity losses due to diabetes, such as the loss of productivity of family members who take care of diabetes patients. These indirect productivity losses increase the estimates of productivity losses. In contrast to other studies, we used disposable income, rather than GDP, to better reflect the urban–rural income gap in our estimate of the cost of productivity losses. We also used the human capital method [22] to estimate the economic impact of death, disability, and unemployment compared to fractional cost estimates in other studies [31,32]. Although suitable to estimate the productivity losses from the employer’s perspective and for a skilled workforce context, the fraction cost approach is less valid in the Chinese context of a large proportion of unskilled workers, especially in the rural sector [1,33]. Finally, consistent with other studies, we do not have age-specific and gender-specific income data or data on those who retire from the workforce before age 69.

## 5. Conclusions

Our key contribution is the provision of the first estimates of productivity losses due to diabetes in rural China and the first comparison of urban–rural productivity differences. The cost of productivity losses for diabetes was significantly higher in urban regions than in rural regions. The results indicate that further investment is required in the prevention, diagnosis, and control of diabetes in under-resourced rural locations and also in urban areas, where most diabetes cases reside. We recommend a targeted approach to the implementation of diabetes prevention and health management measures. For rural areas, increased resources are required to address deficient primary health facilities and improve the training of medical staff. In both urban and rural areas, improved preventative health services, lifestyle programs for weight management, and health education and health management for diabetes programs are required. Such targeted and effective prevention and management actions need to be differentially applied to urban and rural areas. Further health insurance reforms should ensure that both the urban and rural insured population have equal access to diabetes support.

## Figures and Tables

**Table 1:** Population, prevalence, and mortality from diabetes in urban China in 2017.

Age Group (Years)	Population ^a^	Diabetes Prevalence ^b^ (%)	People with Diabetes	Mortality from Diabetes ^c^ (1/100 Thousand)
Male				
20–24	29,049,757	1.49	432,841	0.16
25–29	4,252,913	2.84	635,528	0.49
30–34	35,580,097	4.95	530,143	0.81
35–39	33,383,495	7.89	497,414	1.33
40–44	34,571,602	11.51	515,117	2.75
45–49	39,391,990	15.43	586,941	4.51
50–54	34,077,670	19.09	507,757	16.46
55–59	20,705,097	21.98	308,506	16.01
60–64	21,843,447	23.69	325,467	36.44
65–69	15,928,398	24.00	237,333	58.78
Total	307,184,466	11.77	36,150,668	9.75
Female				
20–24	26,661,408	0.90	239,953	0.07
25–29	40,408,981	1.59	642,503	0.34
30–34	34,884,709	2.68	934,910	0.43
35–39	32,615,291	4.29	1,399,196	0.51
40–44	33,186,893	6.54	2,170,423	1.42
45–49	37,519,417	9.46	3,549,337	2.44
50–54	32,609,223	13.01	4,242,460	8.5
55–59	20,040,049	17.01	3,408,812	8.09
60–64	22,262,136	21.20	4,719,573	25.97
65–69	16,725,728	25.28	4,228,264	51.16
Total	296,913,835	8.60	25,535,431	6.93
Total male and female	604,098,301	10.21	61,686,098	8.37

^a^: Area- and age-specific population estimates were based on China demographic and Employment Statistical Yearbook 2018. ^b^: Age- and gender-specific prevalence of diabetes based on estimates by age and gender from the 2017 International Diabetes Federation Diabetes Atlas (8th Edition). Number of men and women with diabetes calculated based on prevalence of diabetes, but due to rounding of the data, values may not precisely match. ^c^: Mortality data were derived from China Health Statistics Yearbook 2018.

**Table 2:** Population, prevalence, and mortality of diabetes in rural China in 2017.

Age Group (Years)	Population ^a^	Diabetes Prevalence ^b^ (%)	People with Diabetes	Mortality from Diabetes ^c^ (1/100 Thousand)
Male				
20–24	17,680,825	2.99	528,657	0.23
25–29	19,809,466	4.17	826,055	0.55
30–34	18,711,165	5.63	1,053,439	1.13
35–39	17,564,320	7.34	1,289,221	1.51
40–44	19,759,709	9.27	1,831,725	2.92
45–49	25,811,893	11.33	2,924,488	4.66
50–54	255,09,709	13.42	3,423,403	13.02
55–59	16,135,922	15.44	2,491,386	12.79
60–64	19,714,806	17.27	3,404,747	25.41
65–69	15,004,854	18.82	2,823,914	44.87
Total	195,702,670	10.52	20,597,033	9.98
Female				
20–24	15,440,534	1.37	211,535	0.21
25–29	19,364,078	2.19	424,073	0.44
30–34	18,824,029	3.40	640,017	0.56
35–39	16,675,971	5.08	847,139	0.52
40–44	19,008,495	7.26	1,380,017	1.42
45–49	25,412,621	9.86	2,505,684	2.8
50–54	25,424,757	12.69	3,226,402	9.69
55–59	15,922,330	15.49	2,466,369	10.17
60–64	19,118,932	17.96	3,433,760	27.15
65–69	15,307,039	19.86	3,039,978	53.78
Total	190,498,786	9.53	18,174,975	9.87
Total male and female	386,201,456	10.04	38,772,008	9.93

^a^: Area- and age-specific population estimates were based on China demographic and Employment Statistical Yearbook 2018. ^b^: Age- and gender-specific prevalence of diabetes based on estimates by age and gender from the 2017 International Diabetes Federation Diabetes Atlas (8th Edition) Number of men and women with diabetes calculated based on prevalence of diabetes, but due to rounding of the data, values may not precisely match. ^c^: Mortality data were derived from China Health Statistics Yearbook 2018.

**Table 3:** Premature deaths, YPLL, WYLL, and productivity losses due to diabetes for urban workers in 2017.

Age Group (Years)	Premature Deaths Due to Diabetes	YPLL ^a^ (%)	WYLL ^b^ (%)	Productivity Losses ($)
Male				
20–24	46	3138 (0.32)	3138 (1.51)	16,907,102
25–29	209	13,076 (1.32)	13,076 (5.87)	70,455,597
30–34	288	16,607 (1.67)	16,607 (6.85)	89,482,869
35–39	444	23,427 (2.36)	23,427 (8.64)	126,230,345
40–44	951	45,643 (4.60)	45,643 (14.39)	245,939,085
45–49	1777	76,804 (7.74)	76,804 (19.20)	413,847,755
50–54	5609	216,731 (21.86)	216,731 (36.38)	1,167,825,945
55–59	3315	113,348 (11.43)	113,348 (7.17)	610,758,355
60–64	7960	238,172 (24.02)		1,283,354,947
65–69	9363	244,728 (24.68)		1,318,681,817
Total	29,962	991,672 (69.27)	508,772 (89.11)	5,343,483,814
Female				
20–24	19	1093 (0.25)	1093 (3.63)	5,891,021
25–29	137	7366 (1.67)	7366 (21.87)	39,690,845
30–34	150	7296 (1.66)	7296 (18.58)	39,314,072
35–39	166	7266 (1.65)	7266 (14.71)	39,152,446
40–44	471	18,296 (4.16)	18,296 (25.01)	98,585,863
45–49	915	31,083 (7.07)	31,083 (16.20)	167,487,068
50–54	2772	80,768 (18.36)		435,207,634
55–59	1621	39,618 (9.01)		213,478,340
60–64	5781	114,585 (26.05)		617,422,102
65–69	8557	132,546 (30.13)		714,207,692
Total	20,591	439,918 (30.13)	72,401 (10.89)	2,370,437,082
Total male and female	50,552	1,431,591 (100)	581,173 (100)	7,713,920,897

^a^: YPLL: years of potential life lost; ^b^: WYLL: working years of potential life lost.

**Table 4:** Premature deaths, YPLL, WYPLL, and productivity losses due to diabetes for rural workers in 2017.

Age Group (Years)	Premature Deaths Due to Diabetes	YPLL ^a^ (%)	WYLL ^b^ (%)	Productivity Losses ($)
Male				
20–24	41	2513 (0.44)	2513 (0.89)	4,997,584
25–29	109	6224 (1.08)	6224 (2.21)	12,376,873
30–34	211	11,099 (1.93)	11,099 (3.93)	22,070,892
35–39	265	12,694 (2.20)	12,694 (4.50)	25,243,589
40–44	577	25,029 (4.35)	25,029 (8.87)	49,772,781
45–49	1203	46,778 (8.12)	46,778 (16.58)	93,023,433
50–54	3321	114,813 (19.93)	114,813 (40.69)	228,320,655
55–59	2064	63,015 (10.94)	63,015 (22.23)	125,313,517
60–64	5010	134,606 (23.37)		267,682,183
65–69	6733	159,192 (27.64)		316,574,550
Total	19,533	575,961 (53.84)	282,163 (59.81)	1,145,376,058
Female				
20–24	32	2050 (0.42)	2050 (1.08)	4,075,985
25–29	85	4966 (1.01)	4966 (2.62)	9,874,940
30–34	105	5624 (1.14)	5624 (2.97)	11,184,007
35–39	87	4207 (0.85)	4207 (2.22)	8,365,828
40–44	270	11,795 (2.39)	11,795 (6.22)	23,455,292
45–49	712	27,777 (5.63)	27,777 (14.65)	55,237,348
50–54	2464	84,875 (17.19)	84,875 (44.76)	168,785,918
55–59	1619	48,310 (9.79)	48,310 (25.48)	96,071,134
60–64	5191	131,550 (26.65)		261,605,272
65–69	8232	172,558 (34.95)		343,154,307
Total	18,797	493,711 (46.16)	189,603 (40.19)	981,810,031
Total male and female	38,331	1,069,672 (100)	471,766 (100)	2,127,186,089

^a^: YPLL: years of potential life lost; ^b^: WYLL: working years of potential life lost.

**Table 5:** Cost of absenteeism, presenteeism, and labor force dropout in 2017 (USD, billion).

Areas	Absenteeism	Presenteeism	Labor Force Dropout	Total
Urban	22.61 (34.40%)	1.78 (65.41%)	207.28 (77.19%)	231.59 (68.75%)
Rural	43.10 (65.60%)	0.94 (34.59%)	61.24 (22.80%)	105.28 (31.25%)
Total	65.71 (19.51%)	2.71 (0.80%)	268.52 (79.71%)	336.94 (100%)
